# Association of PRLR, IGF1, and LEP genes polymorphism with milk production and litter size in Egyptian Zaraibi goat

**DOI:** 10.1007/s11250-022-03316-2

**Published:** 2022-09-26

**Authors:** Haidan M. El-Shorbagy, Ehab S. Abdel-Aal, Shaimaa A. Mohamed, Akmal A. El-Ghor

**Affiliations:** 1 Zoology Department, Faculty of Science, Cairo University, Giza, 12613 Egypt; 2 Faculty of Biotechnology, October University for Modern Science and Arts, 6th October, Giza, Egypt; 3 Sheep & Goat Research Department, Animal Production Research Institute, Agricultural Research Center (ARC), Giza, Egypt

**Keywords:** PRLR, IGF1, LEP, Goat, Polymorphism, Mutations, Milk production, Litter size

## Abstract

Studying variation in genes responsible for physiological characters is important to enhance goat productive and reproductive efficiency. This study aimed to detect specific nucleotide polymorphisms in prolactin receptor (PRLR), insulin-like growth factor (IGF1), and leptin (LEP) genes and their correlation with milk production (MP) and litter size (LS) traits in Zaraibi goat. PCR-SSCP products of different patterns of each gene were sequenced and aligned to reveal two mutations (T > C) and (G > A) in 3′UTR of PRLR gene and registered on NCBI with accession numbers OM418863 for TT and OM418864 for CT, while (G > A) variation was registered as OM418861 for GG and OM418862 for AG in exon 10. TT, CT, AG, and GG genotypes were distributed in the studied animals with frequencies 0.43, 0.57, 0.65, and 0.35, respectively. While alleles C, T, A, and G frequencies were 0.28, 0.72, 0.32, and 0.68, respectively. CT and AG genotypes associated significantly (*P* < 0.05) with higher MP and LS, respectively. By studying the haplotypes of PRLR, C-A and T-A were associated with the highest and the lowest level of MP, respectively. For LS, T-A and C-G showed significant correlation with the highest and the lowest rate, respectively. Regarding IGF1 gene, two polymorphisms were detected; T74C at exon 4 which registered on NCBI as OM418860, and combined mutations as ins. G470, A531G, and T534C (PP genotype) at 5′ flanking region that registered as OM418859. For LEP, only one polymorphism was found in intron 2 (G281A) which submitted to NCBI as OM418855. All detected polymorphisms have shown to be involved in regulating the MP or LS as reproductive traits in goat.

## Introduction

Goats are among the first domesticated farm animals, due to their great adaptability to different environmental conditions; therefore, they can be raised in arid, humid, tropical, cold, desert, or mountain conditions (Kaliber et al. 2016). Goats are considered very useful animals for their good products and are easy to handle; moreover, they do not compete with man for food. In Egypt, there are five indigenous goat breeds: Baladi (primarily in the delta), Barki (in the west desert), Zaraibi (northeast of the delta), Sinaoy (in the Siani peninsula), and Saidi (in Upper Egypt). Zaraibi goats are dual-purpose animal and considered as being the most promising dairy goat among the local Egyptian breeds due to its high genetic potential for prolificacy and milk production (Dowidar et al. 2018). Small animal holders raised Zaraibi goats as a source for income, which play a role in the development of rural and nomadic communities from an agricultural standpoint (Mohamed 2016).

Although the milk production potential of goat dams is highly associated with their kids’ growth and survival, litter size (LS, numbers of kids born per doe) is also a very important factor determining the reproductive efficiency of the farm animals and has a highly significant influence on goat prolificacy (Tesema et al. 2020). For Zaraibi goat, litter size ranged from 2.1 to 2.14 kids per doe (Abu El-ella et al. 2011; Aboul-Naga et al. 2012), while total milk yield (TMY) ranged from 249 to 363.15 kg/h (El-Saied et al. 2007; Abdelhamid et al. 2013). For milk composition, percentage of fat, protein, lactose, total solid, and solid not fat recorded for Zaraibi goats were 3.31, 2.68, 4.12, 10.73, and 7.5%, respectively (Mohamed 2016). Zaraibi goat’s milk composition is very similar to cow’s milk; it is used as an alternative to cow milk especially for infants because of its digestibility and low allergenicity (Mowlem 2005).

As milk production and reproduction are complex traits (i.e., controlled by many genes and environmental factors), some nucleotides polymorphism might account for large amounts of genetic variation. Therefore, selection programs using specific genetic markers could be a good strategy for precise and improving genetic changes of these traits (Bhowmik et al. 2019). Genes attributed to different economic traits including growth, reproduction, meat, and milk production traits and disease resistance traits are known as candidate genes (Supakorn 2009).

Prolactin plays an important regulatory function in mammary gland development. It is an anterior pituitary peptide hormone and is essential for reproductive performance. The action of this hormone is mediated by its receptor encoded by the prolactin receptor gene (PRLR), which is a member of the growth hormone/prolactin receptor gene family (Ahlawat et al. 2015). In goats, the PRLR gene was mapped on chromosome 20 and consists of five exons and four introns, encoding the 199 amino acid for mature protein (Hayes et al. 1996). PRLR is considered an excellent candidate for linkage analysis of quantitative trait loci (QTL) affecting milk production traits (Shi et al. 2012).

Previous research proved the importance of exon 10 in litter size traits of goats (Zhang et al. 2007; Li et al. 2011; Wu et al. 2014). Also, 3′UTR region is considered an effective region for regulating mRNA-based processes (Mayr 2019). For PRLR, Hou et al. 2014b studied 3′UTR region as a functional region for milk production in goats.

In previous studies, many variations were detected in exon 10, exon 9, and 3′UTR regions of PRLR gene which had a significant effect on milk production and litter size in goats (Zhang et al. 2007; Wu et al. 2014; Hou et al. 2014a, 2014b).

Insulin-like growth factor 1 (IGF1) is considered a strong candidate gene for reproductive traits (Gobikrushanth et al. 2018). In goats, the IGF1 gene was mapped on chromosome 5 (Naicy et al. 2017) and consists of 1–6 exons in different species (Andrade et al. 2008) that produce a polypeptide of 70 amino acids that are highly conserved (Ge et al. 2001). According to polymorphism studies, some researchers promoted the 5′ flanking region as an influential region for reproductive traits by regulation of gene transcription (Eulalia et al. 2006; Mehmannavaz et al. 2010; Naicy et al. 2016; Rasouli et al. 2016). In addition, Othman et al. 2016 found significant effect of mutations at exon 4 and introns 3 and 4 on productive traits in six goats and sheep breeds reared in Egypt.

Regarding IGF-I mutation detection in ovine breeds, various research reported polymorphisms at 5′ flanking region, intron 4, 5′UTR, and exon 3 of IGF-I gene, and they found a significant association between them and litter size, growth traits, body weight, and milk production, respectively (Naicy et al. 2016; Othman et al. 2016; Lestari et al. 2020; Sebastiano et al. 2020).

Leptin (LEP) is synthesized mainly by adipose tissue; it plays the main role in the regulation and control of the productive performance of animals (Kumar et al. 2020). LEP is synthesized as a prohormone (a premature and inactive form of leptin protein); it consists of 167 amino acids, but its mature functional polypeptides consist of 146 amino acids (Marwarha et al. 2012). It is located on chromosome 4 in the ovine genome and consists of three exons and two introns; two exons only are translated into protein (Wallace et al. 2014). Exon 1 is a non-coding part (Buchanan et al. 2002).

For LEP gene polymorphism, exon 3 and intron 2 were studied in different research which had a significant association with productive and reproductive traits in ovine breeds (Liefers et al. 2002; Abousoliman et al. 2020; Singh et al. 2009; Maitra et al. 2014; Abousoliman et al. 2020).

Therefore, this study was designed to detect variations of PRLR, IGF-1, and LEP genes in Zaraibi goats using single-strand conformational polymorphism (PCR-SSCP) analysis and sequencing technology to investigate the potential associations between these polymorphisms with milk production and litter size.

## Materials and methods

### Chemicals

All chemicals used were of analytical grade and were purchased from Sigma Scientific Services Co. and Promega (Cairo, Egypt). All reagents were used according to the necessary health and safety procedures. The molecular kits are listed elsewhere.

### Animals and ethical considerations

One hundred Zaraibi does (mature goat females) were reared in El-Serw experimental station (in Damietta governorate) belonging to the Animal Production Research Institute (APRI), Agriculture Research Center (ARC). Studied animals raised at same farm and kept under same conditions and management. Does were selected according to their parities (kidding seasons) from 2nd to 5th and (24–45 kg) body weight at mating. Animals were fed according to the Nutrient Requirements of Goat (NRC 2007). Zaraibi goats were supplied by a basal ration consisting of 25% concentrate feed mixture (CFM) beside 75% fresh berseem (Egyptian clover) during winter feeding or 50% CFM and 50% berseem hay during summer feeding. Milk yields of all does were recorded during lactation season for daily milk yield (DMY) and total milk yield (TMY) productions. The number of kids born for each doe was recorded for each season to calculate litter size.

Handling and protection of animals used in the study were done according to the recommendations of European Union directive 86/609/EEC (Louhimies 2002) and approved by the Institutional Animal Care and Use Committee (IACUC), Faculty of Science, Cairo University, Egypt with permit number: CU/I/F/47/18.

### DNA extraction and PCR amplification

Ten milliliter of total blood were collected from the jugular vein of each goat using vacuum tubes containing 7.5 mg of EDTA. All blood samples were stored at − 80 °C until DNA extraction. Genomic DNA was extracted from all collected blood samples using the salting out method as described by Miller et al. 1988. The concentrations and purity of the extracted DNA were measured using a spectrophotometer (Eppendorf Biophotometer plus). PCR amplification was performed using Bio-Rad thermal cycler (model C1000) according to PCR conditions (Table 1), using primer list for amplification of PRLR, IGF-1, and LEP genes (Table 2). PCR amplicons were electrophoresed in 1% agarose gels, using 1 × TBE buffer containing 200 ng/ml of ethidium bromide, then visualized under UV light, and photographed by Bio-Rad Laboratories, Hercules, CA, USA.

**Table 1:** PCR conditions of the primer sets of PRLR, IGF-1, and LEP genes

Character	Initial denaturation	Cycles	Denaturation	Annealing	Extension	Final cycle	Reference
For milk production (PRLR-M)	5 min at 95 °C	35 cycles	94 °C for 30 s	50 °C for 30 s	72 °C for 35 s	10 min at 72 °C	Hou et al. 2014b
For litter size (PRLR-L)	5 min at 94 °C	32 cycles	94 °C for 30 s	55 °C for 30 s	72 °C for 30 s	10 min at 72 °C	Zhang et al. 2007
For milk production (IGF1-M)	5 min at 96 °C	35 cycles	94 °C for 40 s	50 °C for 40 s	72 °C for 40 s	10 min at 72 °C	Deng et al. 2010
For litter size (IGF1-L)	2 min at 96 °C	35 cycles	95 °C for 30 s	61 °C for 20 s	68 °C for 60 s	5 min at 68 °C	Naicy et al. 2016
For milk production (LEP-M)	2 min at 94 °C	35 cycles	94 °C for 1 min	55 °C for 1 min	72 °C for 1 min	15 min at 72 °C	Singh et al. 2009
For litter size (LEP-L)	2 min at 95 °C	30 cycles	95 °C for 1 min	55 °C for 1 min	72 °C for 1 min	7 min at 72 °C	Singh et al. 2009

**Table 2:** Primers used for amplification of PRLR, IGF1, and LEP genes

Gene name	Primer name	Sequence (5′-3′)	PCR product size
Prolactin receptor (PRLR)	PRLR-M–F PRLR-M–R	AGTGAGAGTTATGGAAGGATG AAGGTTAAGCAACTGGTCTT	443 bp
	PRLR-L–F PRLR-L–R	AAACCCCCTTGTTCTCTGCTA CCCAACCCAACTGGAGTCTGC	315 bp
Insulin-like growth factor-1 (IGF-1)	IGF1-M–F IGF1-M–R	GCTGGGTGTAGCAGTGAACA GTTGCTTCAGAAGCATAACT	320 bp
	IGF1-L–F IGF1-L–R	GGGTATTGCTAGCCAGCTGGT CCGGGCATGAAGACACACACAT	601 bp
Leptin (LEP)	LEP-M–F LEP-M–R	TGGAGTGGCTTGTCATTTCCTTCT GTCCCTGCTTCTGGCCACCTAACT	400 bp
	LEP-L–F LEP-L–R	AGCAGTCCGTCTCCTCCAAA AGATATTTGGATCACATTTCTG	152 bp

### Single-strand conformation polymorphisms

SSCP analysis detects the changed migration rate of DNA molecules due to sequence-dependent differential intramolecular folding of ssDNA under non-denaturing gel electrophoresis conditions (Orita et al. 1989). Five microliter aliquot of each amplicon was mixed with 5 µl of T.E. buffer and 5 µl of loading dye (98% formamide, 10 mM EDTA, 0.025% bromophenol blue, and 0.025% xylene cyanol). After denaturation at 95 °C for 10 min, samples were rapidly cooled on wet ice for 10 min. Electrophoresis was carried out in 20 ×15 cm, 10% acrylamide: bisacrylamide (29:1) gels (Gasser et al. 2006) at 150 V for 4 h, then stained with ethidium bromide, visualized under UV light, and photographed by Bio-Rad Laboratories, Hercules, CA, USA.

### Sequence analysis

PCR products representing different SSCP patterns of PRLR, IGF-1, and LEP genes were purified and sequenced by automated DNA ABI Prism 3130 Genetic Analyzer using the same sets of primers used for PCR (Sanger et al. 1977). The nucleotide sequences were compared against the corresponding goat gene sequence (GenBank accession number: KC109741.1 and EU662222.1 for PRLR, KX432967.1 and KT315919.1 for IGF-1 gene, and EU220012.1 and MH716185.1 for LEP) and analyzed by Clustal Omega and Jalview sequence alignment editor program 2.11.1.6. The nucleotide sequences of new gene variants were submitted to NCBI and approved for publishing.

### Statistical analysis

Analysis of variance and least squares means were calculated using the general linear model (GLM) procedure of SAS (2004). Three models were performed; the first one was used to estimate the effect of all parameters (parity, breeding season, PRLR-M genotypes, PRLR-L genotypes, and haplotypes PMPL) on ADMY, while the second and third models were explored the effect of the same parameters on TMY and litter size, respectively.

*Y*_*ijklmn*_: *µ* + *P*_*i*_ + *S*_*j*_ + *PM*_*k*_ + *PL*_*l*_ + *PMPL*_*m*_ + *e*_*ijklmn*_*.*

where.

μ: the overall population mean,
*Y*: the observed records of ADMY, TMY, and LS,
*P*_*i*_: the fixed effect of i^th^ parity of does (*i* = 1,…5),
S_j_: the fixed effect of the j^th^ breeding season (*j* = autumn, spring),
*PM*: the fixed effect of k^th^ PRLR-M genotypes (*k* = CT, TT),
*PL*: = the fixed effect of l^th^ PRLR-L genotypes (*l* = AG, GG),
*PMPL*: the fixed effect of m^th^ haplotypes of PRLR-M and PRLR-L (*m* = C-A, C-G, T-A, T-G),
*e*_*ijklmn*_:  = random error.

Tukey–Kramer test was used to compare the least squares mean of the genotypes. Genetic equilibrium was estimated according to the Hardy–Weinberg equilibrium (HWE) and chi-square test using Michael H. Court’s (2005–2008) calculator.

## Results

A total of 100 Zaraibi does genotype for polymorphisms in 3′UTR and exon 10 in PRLR, exon 4 and 5′ flanking region in IGF-1, and intron 2 and exon 3 in LEP. The PCR products successfully amplified as follow: 443 bp fragment of 3′UTR flanking region 1–443 bp and 315 bp of exon 10 of PRLR gene flanking region 424–738 bp (GenBank accession number: KC109741.1 and EU662222.1, respectively). For IGF-1 gene, 320 bp fragment of exon 4 flanking region 1–320 bp and 601 bp of 5′ flanking region 1–601 bp (GenBank accession number: KX432967.1 and KT315919.1, respectively), while 400 bp of intron 2 flanking region 1–400 bp and 152 bp of exon 3 flanking region 779–930 bp of LEP gene (GenBank accession number: EU220012.1 and MH716185.1, respectively).

### PCR-SSCP analysis

**Fig. 1:**
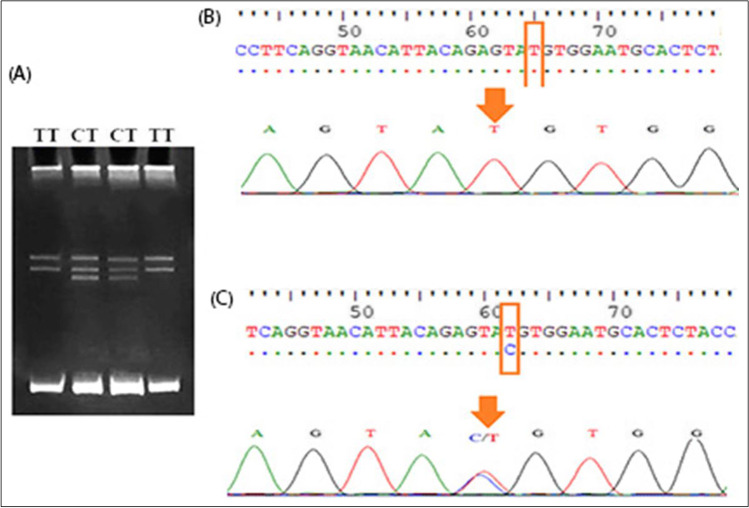
Genotyping of 3′ UTR fragment of PRLR gene. **A** The electrophoretic pattern obtained after SSCP analysis shows homozygous genotype TT (lanes 1, 4) and heterozygous genotype CT (lanes 2, 3). **B** DNA sequencing analysis representing TT genotype. **C** DNA sequencing analysis representing CT genotype

**Fig. 2:**
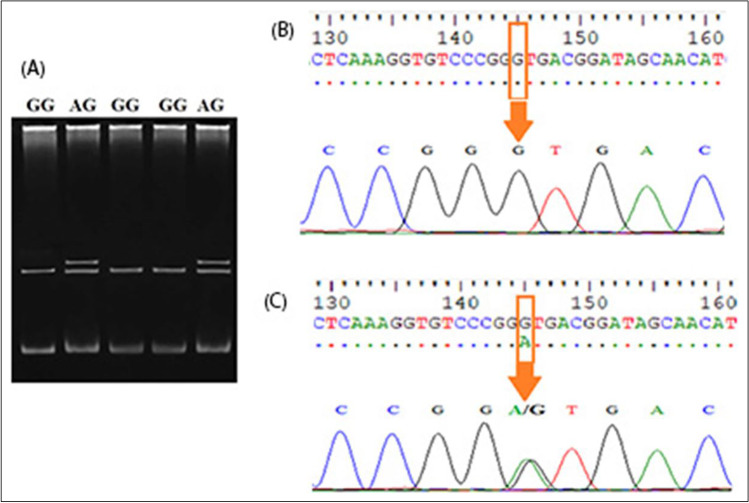
Genotyping of exon 10 regions of PRLR gene. **A** The electrophoretic pattern obtained after SSCP analysis shows homozygous genotype GG (lanes 3, 4) and heterozygous genotype AG (lanes 2, 5). **B** DNA sequencing analysis representing GG genotype. **C** DNA sequencing analysis representing AG genotype

**Fig. 3:**
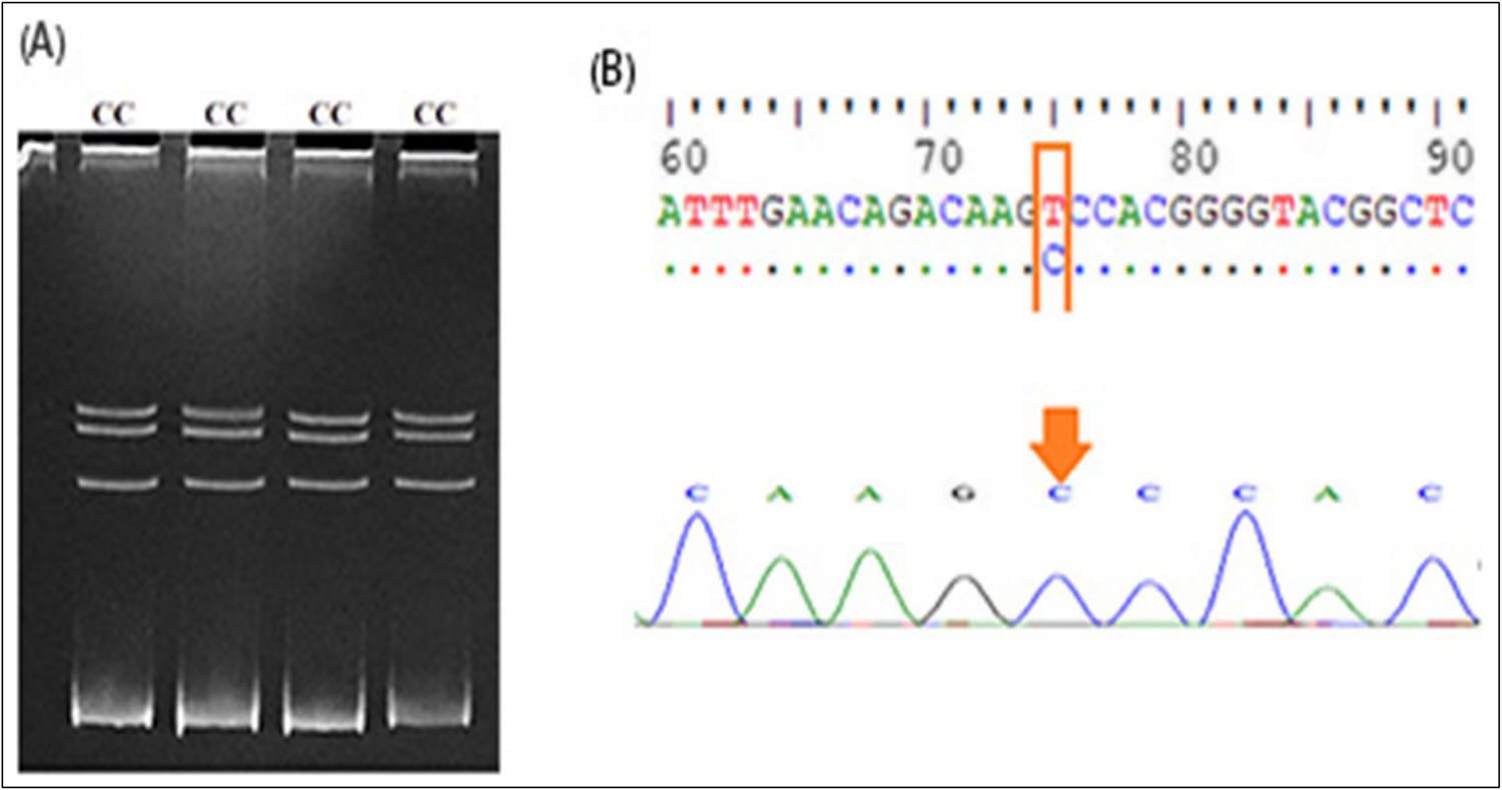
Genotyping of exon 4 of the IGF-1 gene. **A** The electrophoretic pattern obtained after SSCP analysis shows homozygous genotype CC (lanes 1–4). **B** DNA sequencing analysis representing CC genotype

**Fig. 4:**
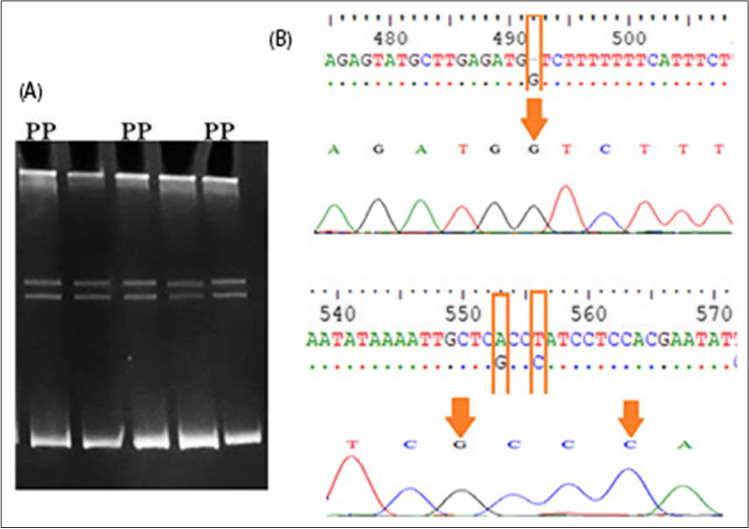
Genotyping of 5′ flanking region of IGF-1 gene. **A** The electrophoretic pattern obtained after SSCP analysis showing homozygous genotype PP (lanes 1–3). **B** DNA sequencing analysis representing PP genotype (ins. G at 471, A530G, and T533C)

**Fig. 5:**
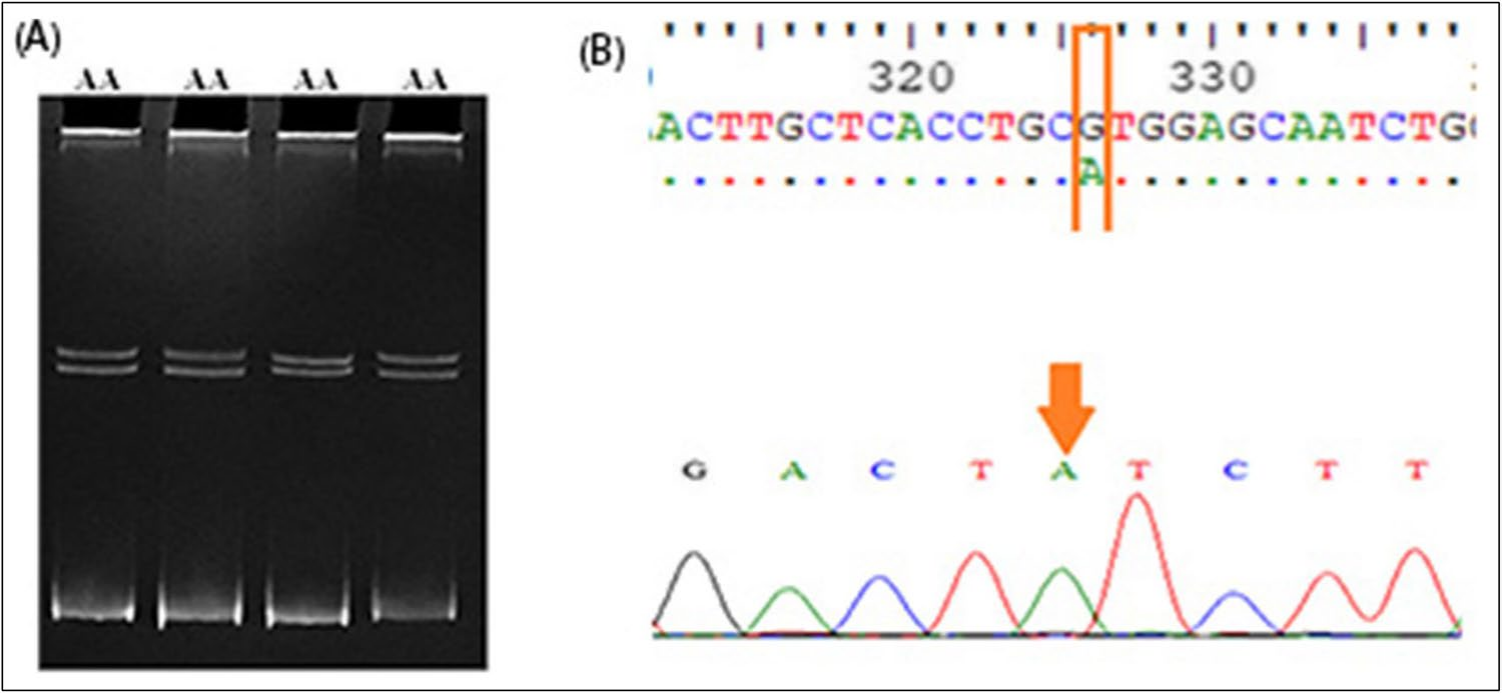
Genotyping of intron 2 regions of LEP gene. **A** The electrophoretic pattern obtained after SSCP analysis shows homozygous genotype AA (lanes 1–4). **B** DNA sequencing analysis representing AA genotype

Regarding the PRLR gene, PCR-SSCP analysis revealed two genotypes (TT and CT) for the 3′UTR region with frequencies of 0.43 and 0.57, respectively, and allelic frequencies of 0.28 and 0.0.72 for C and T alleles, respectively (Fig. 1A) (Table 3). Another two genotypes (AG and GG) were observed at exon 10 with frequencies of 0.65 and 0.35, respectively, and allelic frequencies of 0.32 and 0.68 for A and G alleles, respectively (Fig. 2A) (Table 3). For IGF-1 gene, two genotypes were detected as CC genotype (for exon 4, Fig. 3A) and PP (5′ flanking region (Fig. 4A) which contains three mutations ins. (G) at position 470, A531G, and T534C. However, in the LEP gene, only the AA genotype was observed in intron 2, and there is no variation in exon 3 (Fig. 5A).

**Table 3:** Genotype and allelic frequencies of the PRLR locus in Zaraibi goats

	Genotypes’ frequencies		Allelic frequencies			
PRLR-M	CT	TT	C	T	*χ*^2^	*P*-value
	0.57 (57)	0.43 (43)	0.285	0.725	15.89	*P* < 0.01
PRLR-L	AG	GG	A	G		
	0.65 (65)	0.35 (35)	0.32	0.68	23.18	*P* < 0.01

CT, TT, AG, and GG genotype frequencies were at the PRLR locus; *n* = 100 Zaraibi does; genotypes and alleles frequencies were assessed according to Hardy–Weinberg equilibrium (HWE) and *χ*^2^, chi-square value. The number of animals per genotype is indicated in parentheses

Genetic equilibrium was estimated based upon the Hardy–Weinberg equilibrium and chi-square test using. The obtained results showed that genotype distributions of two loci in the PRLR gene disagree with the Hardy–Weinberg equilibrium (*P* < 0.05) for the Zaraibi goat breed which proves that the genotypic frequencies had been affected by selection and mutation (Table 3).

### Sequencing analysis

After sequencing 3′UTR in the PRLR gene, one polymorphism was detected with nucleotide transition from thymine (T) to cytosine (C) at position 62 when compared with the PRLR gene (accession number: KC109741.1) and indicated in our sequence deposited in the nucleotide database with the accession numbers OM418863 and OM418864 (Fig. 1B, C). For exon 10, the transition from guanine (G) to adenine (A) at position 567 was found by comparing it with the PRLR gene (acc. no. EU662222.1) and indicated in our sequence deposited in the nucleotide database with the accession numbers OM418861 and OM418862; this mutation resulted in an amino acid change from valine (V) to methionine (M) (Fig. 2B, C).

For IGF1, after comparing nucleotide sequence with IGF1 gene (acc. no. KX432967.1), one point mutation was observed in exon 4 at position 74 from T to C; this variation submitted to GenBank with accession number OM418860 which led to a change of amino acid from serine (S) to proline (P) (Fig. 3B). Another three polymophisms (ins. (G) at position 470, A531G, and T534C) were found in the 5′ flanking region by comparing the sequence with acc. no. KT315919.1 and registered in GenBank with accession number OM418859 (Fig. 4B).

However, the LEP gene had one variation (G281A) in intron 2 when compared with GenBank (acc. no. EU220012.1) and was submitted to GenBank with accession number OM418855 (Fig. 5B, C). On the other hand, exon 3 did not have any variation from the sequence on GenBank (acc. no. MH716185.1). All previous mutations have been recorded for the first time in this study and submitted to GenBank as indicated previously.

Because of there is only one genotype in each region of IGF-I and LEP genes, association of this genotype with MP and LS could not be studied.

### Effect of PRLR genotypes on MP and LS

As shown in the Table 4, at 3′UTR of PRLR gene (PRLR-M), the transition of T>C at position 62 has a highly significant effect on ADMY and TMY. CT genotype has higher ADMY (0.86 ± 0.03 kg) and TMY (211.88 ± 6.8 kg) than TT (0.79 ± 0.03 and 182.15 ± 7.6, respectively). However, in exon 10 (PRLR-L), the A>G mutation at position 567 significantly affected the LS of does, where the AG genotype has a higher LS (2.10 ± 0.1 kids) than the GG genotype (1.79 ± 0.1 kids).

**Table 4:** Effect of PRLR genotypes on average daily, total milk yield, and litter size

Genotypes	Average daily milk yield (kg/day)	Total milk yield (kg)	Litter size (kids)
PRLR-M			
CT	0.86 ± 0.03 ^a^	211.88 ± 6.8 ^a^	2.04 ± 0.1^a^
TT	0.79 ± 0.03 ^b^	182.15 ± 7.6 ^b^	2.10 ± 0.1^a^
PRLR-L			
AG	0.84 ± 0.03 ^a^	203.46 ± 7.2 ^a^	2.10 ± 0.1^a^
GG	0.83 ± 0.03 ^a^	212.46 ± 8.3 ^a^	1.79 ± 0.1^b^

Results were expressed as least squares means (LSM) ± standard error (SE); mean values marked with the different letters are different (significant,* P* < 0.05)

For haplotype distribution frequencies, it was found that C-A is the most frequent haplotype at 35%, followed by T-A (30%), then C-G (22%), and T-G is the least frequent (13%). Meanwhile, these haplotypes had a significant effect on ADMY, TMY, and LS (*P* < 0.01); the results indicated that C-A has the highest level of ADMY and TMY (0.87 ± 0.03 and 220.64 ± 7.0, respectively), while T-A has the lowest level (0.73 ± 0.03 and 181.75 ± 7.9). However, for litter size, T-A has the highest level (2.11 ± 0.1 kids), and C-G was the lowest one (1.76 ± 0.1 kids) (Table 5).

**Table 5:** Effect of haplotype on average daily, total milk yield, and litter size

	Average daily milk yield (kg/ day)	Total milk yield (kg)	Litter size (kids)
C-A	0.87 ± 0.03 ^a^	220.64 ± 7.0 ^a^	2.10 ± 0.1^a^
C-G	0.84 ± 0.03 ^ab^	217.56 ± 8.5 ^a^	1.76 ± 0.1^b^
T-A	0.73 ± 0.03 ^b^	181.75 ± 7.9 ^b^	2.11 ± 0.1^a^
T-G	0.78 ± 0.04 ^b^	187.21 ± 9.4 ^b^	1.84 ± 0.1^b^

Results were expressed as least squares means (LSM) ± standard error (SE); mean values marked with the different letters are different (significant,* P* < 0.05).

### Effect of parity and breeding season on MP and LS

The overall means of ADMY, TMY, and LS were 0.80 kg, 198.7 kg, and 1.96 kids, respectively. ADMY, TMY, and LS of Zaraibi does were affected significantly by parity (*P* < 0.01); they showed the lowest value at the first parity (0.74 ± 0.03, 179.62 ± 7.3 kg, and 1.76 ± 0.1 kids, respectively) and increased gradually until highest level at the 4th parity (0.87 ± 0.04, 214.14 ± 9.4 kg, and 2.11 ± 0.1 kids, respectively) (Table 6). The breeding season significantly affected (*P* < 0.05) the ADMY and TMY, where does produce higher amount of milk in spring than in autumn.

**Table 6:** Effect of parity and breeding season on average daily, total milk yield, and litter size

Items	Average daily milk yield (kg/day)	Total milk yield (kg)	Litter size
Parity			
1	0.74 ± 0.03^b^	179.62 ± 7.3^c^	1.76 ± 0.1^c^
2	0.85 ± 0.03^a^	201.35 ± 7.9^ab^	1.85 ± 0.1^bc^
3	0.86 ± 0.03^a^	208.31 ± 8.5^a^	1.99 ± 0.1^ab^
4	0.87 ± 0.04^a^	214.14 ± 9.4^a^	2.11 ± 0.1^a^
≥ 5	0.78 ± 0.04^b^	193.03 ± 10.6^bc^	2.06 ± 0.1^a^
Breeding season			
Autumn (November)	0.84 ± 0.03^b^	197.36 ± 8.0^b^	1.96 ± 0.1^a^
Spring (March)	0. 90 ± 0.03^a^	201.22 ± 7.1^a^	1.94 ± 0.1^a^

Results were expressed as least squares means (LSM) ± standard error (SE); mean values marked with the different letters are different (significant, *P* < 0.05)

## Discussion

In recent years, a great interest is focused on using molecular markers in understanding the animal genome and genetic diversity analysis. Molecular markers have been widely used to assess genetic variations since they provide information on every region of the genome. In addition, polymorphism in the transcription factor binding sites is important, as nucleotide substitutions may change the level of gene expression (Ge et al. 2001). This study evaluated the correlation of some newly detected polymorphisms in candidate genes (PRLR, IGF1, and LEP) with productive and reproductive traits for the Zaraibi goat.

PCR-SSCP and sequence analysis concluded two genotypes (TT and CT) in 3′UTR of PRLR gene with significant associated with milk production where CT has a greater milk yield. This is in harmony with the findings of Hou et al. (2014b), who found that CC and CT genotypes were significantly associated with milk production, while CC has a greater milk yield in Chinese goat breeds.

Regarding exon 10 in the PRLR gene, it has two genotypes (AG and GG) with a significant effect on litter size where AG has a stronger effect than GG; these results are in harmony with that reached by Zhang et al. (2007) who noticed FF, FG, and GG genotypes with higher litter size for FG does (*P* < 0.05) in Chinese goat breeds. Also, Wu et al. (2014) investigated exon 10 of the PRLR gene and found four genotypes (AA, AB, AC, and AD) with a significant effect on litter size in Lezhi black goats, and he has concluded that PRLR gene expression and mutations in exon 10 of PRLR gene may be associated with the reproductive effects of goat. Hou et al. (2013) screened four novel mutations: g.40452 T > C and g.40471G > A mutations were in the intron 2, and g.61677G > A and g.61865G > A mutations were in the exon 9 in PRLR gene with significant association with milk production traits in Saanen and Guanzhong goat breeds. In addition, Gao et al. (2015) studied polymorphisms at exon 10 of the PRLR gene and found three genotypes (BB, BC, and CC) in one locus and DE and DD in another locus in Chinese sheep breeds that did not significantly affect the litter size.

For exon 4 at IGF-1, one genotype CC was found; this result agreed with Deng et al. (2010) who reported a higher significant effect on milk yield for the CC genotype than other ones. In the current study, one genotype (PP) was observed in the 5′ flanking region of the IGF1 locus. Also, Sebastiano et al. (2020) found that the AA genotype in exon 3 showed higher milk yield in Sarda dairy sheep. However, in the Markhoz goat, Rasouli et al. (2016) noticed 3 genotypes (GG, AG, and AA) with significant association with litter size.

In the LEP gene, we recorded one genotype (AA) in intron 2, and we did not find any variation in exon 3 between Zaraibi does. On the other hand, three genotypes in exon 3 were found by Abousoliman et al. (2020) with significant association with milk yield in Barki sheep. On the other hand, Bhowmik et al. (2019) proved that reproductive traits of the cow were associated with the T allele of the LEP gene, and 3 genotypes (CC, CT, and TT) were reported with a significant effect on litter size.

In the present study, ADMY and TMY were significantly affected by parity (*P* < 0.01) and breeding season (*P* < 0.05), where they increased gradually with the increase of parity until the fourth parity. Means of the ADMY and TMY were higher in March (spring) compared with November (autumn); this may be due to good nutrition and availability of Egyptian clover. These findings agreed with Hamed et al. (2009) who found that ADMY and TMY of Zaraibi goats were significantly affected by parity. The same results were estimated by Mohamed (2016) who concluded that milk yield increased with the raising of the parity number. In addition, litter size was significantly affected by parity (*P* < 0.05), where it progressed from the first parity until the fourth parity, which may be due to the improvement of reproduction efficiency in the goat farm because of the management system permitted to cull does with low litter size. This result agreed with Hamed et al. (2009) who reported that year and breeding season have a significant effect on litter size in Zaraibi goats. On the contrary, breeding season has no significant effect on Zaraibi goat litter size.

These results provide new insights into the crucial role of genetics polymorphism on productive and reproductive performances in goats, where genotype/phenotype association has become an important reference to be used in goat breeding programs.

## Conclusion

PRLR, IGF-I, and LEP could be considered potential candidate gene markers for productive and reproductive traits; their polymorphisms were investigated in dairy and prolific Zaraibi goats. All polymorphisms investigated be involved in regulating milk production and reproductive activity. So, these variations can be used successfully in marker-assisted selection programs to select individual with favorable characters which improve the productive and reproductive performances of goats’ herd and could be identified before using a successful breeding program. It will be interesting to conduct further researches on a higher number of animals and other regions in gene to confirm the effect of the found mutations and to highlight other polymorphisms in the sequence of these genes that can also be associated with productive and reproductive traits in goat.

## Data Availability

All data analyzed during the current study are available from the corresponding author on request.
